# A Novel Mutation in the Transglutaminase-1 Gene in an Autosomal Recessive Congenital Ichthyosis Patient

**DOI:** 10.1155/2014/706827

**Published:** 2014-08-10

**Authors:** D. Vaigundan, Neha V. Kalmankar, J. Krishnappa, N. Yellappa Gowda, A. V. M. Kutty, Patnam R. Krishnaswamy

**Affiliations:** ^1^Genomics and Central Research Laboratory, Department of Cell Biology and Molecular Genetics, Sri Devaraj Urs Academy of Higher Education and Research, Tamaka, Kolar 563101, India; ^2^Molecular Biophysics Unit, Indian Institute of Science, Bangalore 560012, India; ^3^National Centre for Biological Sciences, Bangalore 560065, India; ^4^Department of Paediatrics, Sri Devaraj Urs Medical College, Tamaka, Kolar 563101, India

## Abstract

Structure-function implication on a novel homozygous Trp250/Gly mutation of transglutaminase-1 (TGM1) observed in a patient of autosomal recessive congenital ichthyosis is invoked from a bioinformatics analysis. Structural consequences of this mutation are hypothesized in comparison to homologous enzyme human factor XIIIA accepted as valid in similar structural analysis and are projected as guidelines for future studies at an experimental level on TGM1 thus mutated.

## 1. Introduction

Autosomal recessive congenital ichthyosis (ARCI, OMIM 242300) is a heterogeneous group of disorders of keratinization characterized primarily by abnormal skin scaling over the whole body. These disorders are limited to the skin, with approximately two-thirds of patients presenting severe symptoms. The main skin phenotypes are lamellar ichthyosis (LI) and nonbullous congenital ichthyosiform erythroderma (NCIE), although phenotypic overlap within the same patient or among patients from the same family can occur. Neither histopathologic findings nor ultrastructural features clearly distinguish between NCIE and LI [1, 2].

Autosomal recessive congenital ichthyosis (ARCI) is caused by mutation in the genes, encoding keratinocyte transglutaminase (TGM1; 190195) on chromosome 14q11.2, ALOX12B gene (OMIM603741) on chromosome 17p13.1, ALOXE3 gene (OMIM607206) on chromosome 17p13.1, ABCA12 gene (OMIM607800) on chromosome 2q35, CYP4F22 gene (OMIM611495) on chromosome 19p13, NIPAL4 gene (ichthyin; OMIM609383) on chromosome 5q33, LIPN gene (OMIM613924) on chromosome 10q23, CERS3 gene (OMIM615276) on chromosome 15q26, and PNPLA1 gene (OMIM612121) on chromosome 6p21. Of all the genes, at least one-third of ARCI cases are caused by mutations in transglutaminase-1 (TGM1) gene [3–9].

TGM1 encodes for the transglutaminase-1 (TGase-1) enzyme, which has 817 amino acid residues and a molecular weight of ~89 kD. TGase-1 is a member of a class of enzymes that form *ε*-(*γ*-glutamyl)lysine isopeptide bond cross-links between proteins, thereby forming stable, insoluble barrier in stratified squamous epithelia, in particular in the formation of the cornified cell envelope, which is a 15 nm thick layer of protein deposited just inside the cell periphery.

The active TGase-1 resides in a proteolytically processed form of 67/33/10 kDa chains that are held together by secondary interactions while bound to the membrane through acyl myristate and palmitate adducts on the 10 kDa portion. Mutations in the TGM1 gene, which produce a defective enzyme due to truncations, point substitutions, and so forth, are located on a number of sites along the protein. Patients can be either homozygous for a single mutation or compound heterozygous for two different mutations [10–15]. To date, over 140 different mutations have been identified in TGM1 gene. Here, in this report, we present a novel Trp 250 to glycine mutation of TGM1 gene.

## 2. Materials and Methods

### 2.1. Patient and Phenotypic Features

**Figure 1:**
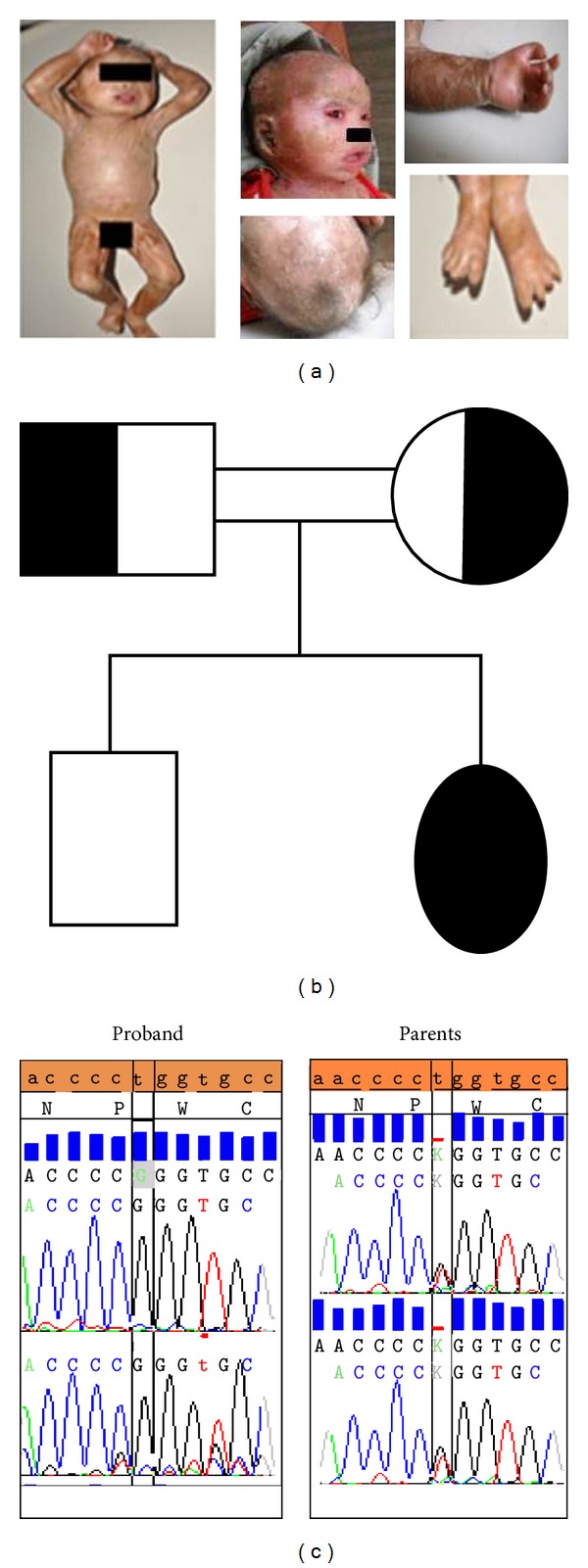
(a) Clinical feature of the proband. (b) Family Pedigree. (c) NG_007150.1:g. [7708T>G] Mutation of TGM1.

An eleven-month-old female child, the second child of a consanguineous marriage (Figure 1(b)), presented with skin lesions since birth. The skin of the baby was thick, gelatinous hard, and parchment-like, strikingly resembling a collodion membrane. The skin scaling was present with fissuring, eyes were wide open, and lids were everted indicating ectropion. The baby's lips were everted indicating eclabium; the scalp showed alopecia; the nose appeared small; the skin of the neck and trunk had multiple deep fissures. The hands presented a form and shape of a claw with limitation of joint movements; digits were small; feet and hands were edematous (Figure 1(a)). The cardiovascular and abdominal examination showed no abnormalities. Further investigations revealed leukocytosis, positive for C-reactive protein, and chest X-ray showed bilateral nonhomogenous opacities. Subsequently, at 14 months of age, the baby expired.

### 2.2. Mutation Analysis

The Institutional Ethical Committee approved the study. Informed consent was obtained from the parent for genetic analysis. Blood samples of the proband and parents were collected for both karyotyping and molecular genetic analyses. Karyotyping of proband was found to be normal. For mutational analysis, the genomic DNA of both proband and parents was isolated and used as a template for the PCR. All the translated exons of TGM1, including the exon-intron boundaries, were amplified by Pfu polymerase. The purified PCR products were sequenced by ABI-3500 genetic analyzer and sequences were analyzed by KB-3500 base caller of ABI Sequence Scape software using the accession number NG_007150 sequence as a template. Further, the impact of mutation was tested with PolyPhen-2 [16].

### 2.3. Conserved Residue Analysis

Protein sequence of the human TGM1 was compared with homologous sequences available using the NCBI BLASTP program using the default database, that is, nonredundant protein sequence database (set of all GenBank CDS translations along with all Refseq, Swiss-Prot, PDB, PIR, and PRF proteins) [17]. From the blast result, 150 homologues of TGM1 were identified. Multiple sequence alignment of all the transglutaminase sequences was performed using the ClustalX program [18]. Conservation of residues across the aligned data set was examined by means of an index of variability. The number “*n*” refers to the number of different amino acids present at each position along the sequence. “*n* = 1” refers to a completely conserved residue in each enzyme dataset, while “*n* = 20” corresponds to a position where all 20 amino acids have occurred at least once.

### 2.4. Structural Analysis

At present, the complete crystal structure of human transglutaminase-1 is not available in the Protein Data Bank (PDB) and only a partial structure of 102 residues (PDB code 2XZZ) containing the beta-barrel domain is available. However, this domain does not include the site of mutation currently being reported. The PSI-BLAST search for TGase-1 against PDB was carried out. The 1GGT crystal structure of human factor XIIIA was used as a model to study the effect of the mutation.

## 3. Results and Discussion

### 3.1. Mutational Analysis

From the mutational analysis of TGM1, the proband was confirmed to be a homozygous T>G transversion in the exon 4 region with the genotype, NG_007150.1:g. [7708T>G]; [7708T>G] (Figure 1(c)). The parents are heterozygous for this mutation (Figure 1(c)). The T>G transversion leads to a mutational change in the amino acid residue Trp250/Gly. Further, the PolyPhen-2 predicted Trp250 substitution glycine might be “probably damaging” with a score of 1.000 (sensitivity: 0.00; specificity: 1.00). The potential effect of the remaining 18 amino acids was also analyzed. All the 18 substitutions were predicted to be “probably damaging” with a score of 1.000. Furthermore, genes known for alopecia associated with neonatal ichthyosis, namely, ATP-binding cassette subfamily A member 12 (ABCA12), membrane-bound transcription factor peptidase, site 2 (MBTPS2), and claudin-1 (CLDN1), were also analyzed by Next Generation Sequencing method. However, no mutations were seen in these genes (data not shown).

### 3.2. Sequence Diversity Analysis

On the basis of the occurrence of the most abundant amino acid at each position and the number of observed replacements, the extent of conservation of the residue position is examined from the dataset of aligned protein sequences. For example, a fully conserved residue is labelled as an *n* = 1 residue, whereas *n* = 2 residue refers to position where two amino acids are seen in the dataset, with the more abundant residue occurring in greater than 90 percent of the sequences. This classification is useful for the analysis of sequence data sets where a large number of sequences (>200) from diverse sources are available. Trp250 of TGM1 was found to be one of the highly conserved residues with *n* = 2, 149/151.

### 3.3. Structural Consequence of Mutation

**Figure 2:**
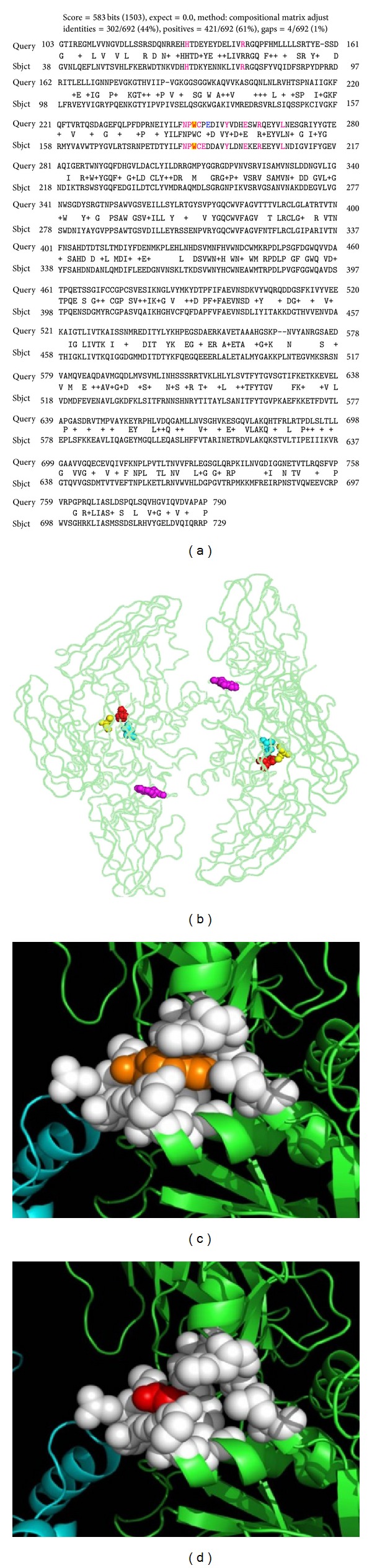
(a) PSI-BLAST of TGM1 against 1GGT structure of human factor XIIIA, Trp 250 highlighted in yellow with its four angstrom neighbor residues. (b) Factor XIIIA structure 1GGT showing active site residues Cys 314 (yellow), His 373 (red), Asp 396 (cyan), and Trp187 (pink). (c) Structural analysis showing 1GGT Trp187. (d) The possible effect of Trp187Gly (p.W250G in TGM1) mutations.

From PSI-BLAST analysis, it was found that the sequence identity between the TGM1 sequence (817 residues) and the human factor XIIIA (PDB code 1GGT) structure (732 residues) was 44% with 84% query coverage (Figure 2(a)). Human factor XIIIA plays a critical role in the blood coagulation cascade. Belonging to the transglutaminase family, it performs cross-linking of soluble fibrin clots into insoluble fibrin clots by a transamidation reaction. It exists as a homodimer (A_2_) intracellularly and also as a heterotetramer (A_2_B_2_) extracellularly. The active site residues comprise the catalytic triad Cys314, His373, and Asp396 (Figure 2(b)). Apart from these key residues, a crucial Trp279 that stabilizes the transition state is also considered as a part of the active site [19]. The spatially proximal residues (within 4 Å) near Trp187 (structurally equivalent to Trp250 in TGM1) were also almost conserved in case of 1GGT and hence were utilized for the possible consequences of the mutation. The key change on this mutation would be loss of packing, since there is compact packing with Trp187 (Figures 2(c) and 2(d)).

**Figure 3:**
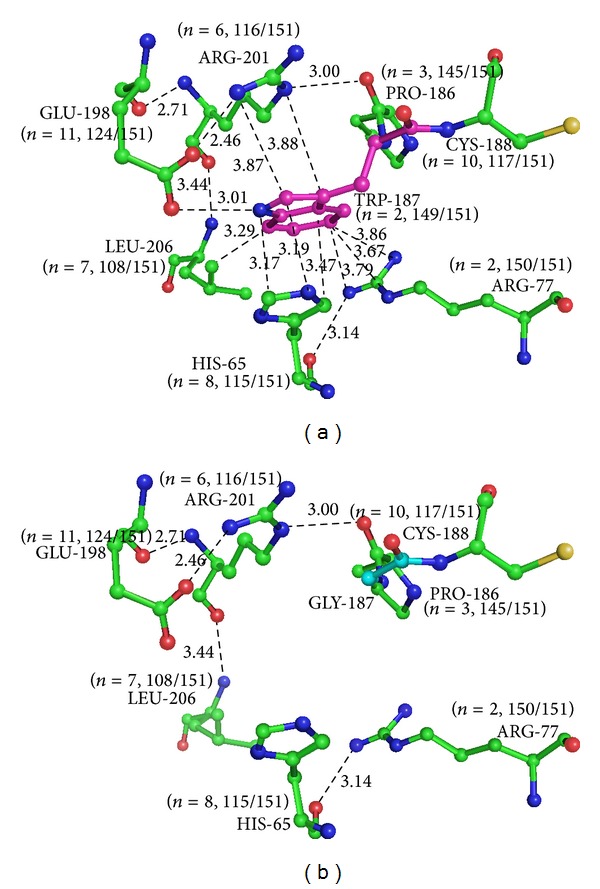
(a) Trp187 (pink) from human factor XIIIA and its proximal interacting residues (green) (within 4 Å) (PDB code 1GGT). (b) Gly187 (cyan) from human factor XIIIA and its proximal interacting residues (green) (within 4 Å) (PDB code 1GGT). The conservation for each residue has been represented in parenthesis with the number of replacements at a position and the number of occurrences in the dataset.

Analysis of the 1GGT structure revealed that the bulky side chain of Trp187 (Trp250 in TGM1) is present in the loop region formed by highly conserved residues, indicating that the local environment of Trp187 has probable structural consequences. It interacts with many highly conserved residues. Trp187 (Trp250 in TGM1) makes the following close contacts: (a) Guanidino-NH group of Arg77 (Arg142 in TGM1) and Arg201 (Arg264 in TGM1) with an aromatic ring of Trp187 is a potential *π*-cationic [20] interaction (3.7–3.9 Å and 3.9 Å, resp.), (b) side chain of Leu206 (Leu269 in TGM1) with the aromatic ring of Trp187 by a nonpolar contact (3.3 Å), (c) gamma carboxyl group of Glu198 (Glu261 in TGM1) with the indole-NH group of Trp187, forming a hydrogen bond (3.0 Å), and (d) imidazole ring of His65 (His130 in TGM1) with the aromatic ring of Trp187 (with least distance of 3.17 Å), a potential example of a parallel *π*-stacking [21] interaction (Figure 3(a)). When Trp187 (Trp250 in TGM1) is mutated to Gly, all these hydrogen-bond and pi-pi and pi-salt bridge interactions are lost (Figure 3(b)). It is also noteworthy that the Arg 142 of TGM1 (Arg77 in 1GGT) has been shown to be a mutation hotspot, and change in Arg 264 of TGM1 (201 in 1GGT) in TGM1 also leads to disease condition.

**Figure 4:**
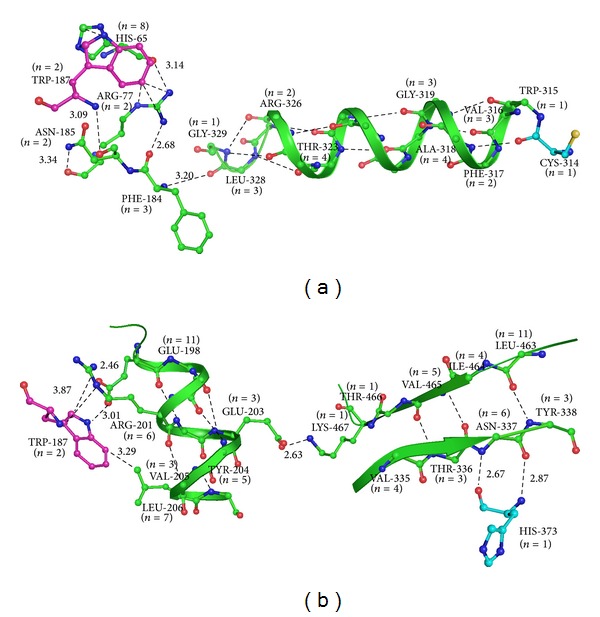
(a) Conserved residue network, between Trp187 (pink) and catalytic residue Cys314 (cyan). (b) Conserved residue network, between Trp187 and catalytic residue His373. The number of replacements at a position has been represented in parenthesis. (PDB code 1GGT).

The site of mutation Trp187 to Gly is >25 Å from active site residues (Cys314, His373, Asp396, and Trp279). The 1GGT structure of human factor XIIIA was examined to seek links between Trp187 and the active site residues, namely, Cys314 (Cys376 in TGM1), His373 (His436 in TGM1), Asp396 (Asp459 in TGM1), and Trp279 (Trp341 in TGM1). From our analysis, we found conserved residue networks from Trp187, leading to catalytic Cys314 (Figure 4(a)) and His373 (Figure 4(b)). Drastic effect on the structure due to p. 250Trp>Gly mutation probably distorts these conserved networks and consequently the function of enzyme (Figure 4).
